# Supplementary figures and images for: Leiomodin creates a leaky cap at the pointed end of actin-thin filaments
Source: PLoS Biol. 2020 Sep 8;18(9):e3000848. doi: 10.1371/journal.pbio.3000848 (PMC7500696; doi:10.1371/journal.pbio.3000848)

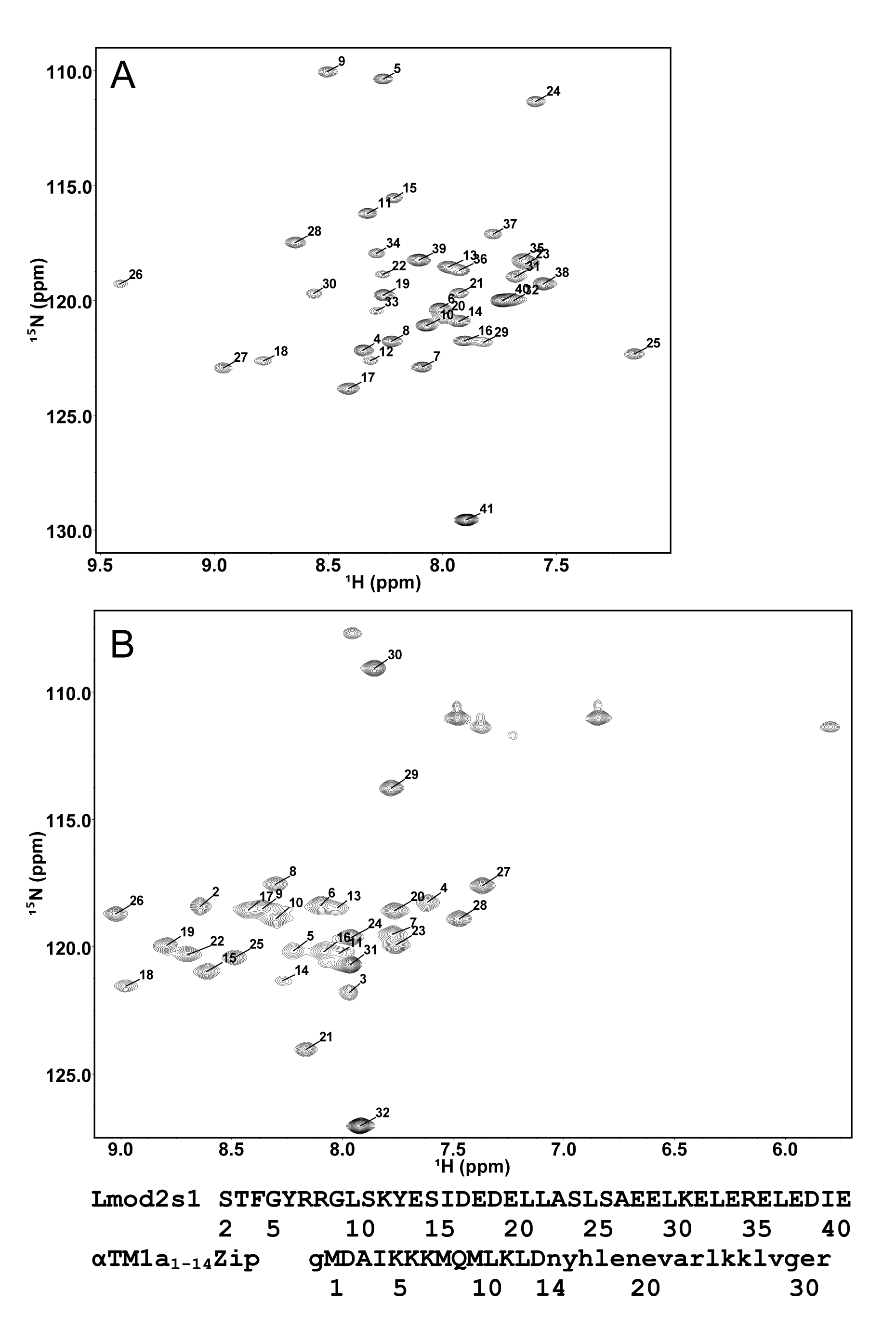

Supplement: S1 Fig — Sequence-specific assignment of 2D 15N-HSQC spectra of (A) 15N/13C-labeled Lmod2s1 in a complex with unlabeled αTM1a1-14Zip, and (B) 15N/13C-labeled αTM1a1-14Zip in a complex with unlabeled Lmod2s1. The spectra were recorded in 50 mM sodium phosphate buffer (pH 6.5), 10% D2O, 0.2 mM EDTA, 0.1% sodium azide, 2X Pierce EDTA-free protease inhibitor cocktail on a Varian 500-MHz spectrometer at 25°C. Sequences of the Lmod2s1 and αTM1a1-14Zip peptides used in the NMR studies are shown at the bottom. The N-terminal Gly and the GCN4 sequence in αTM1a1-14Zip are shown in small letters. 2D, two-dimensional; HSQC, heteronuclear single-quantum coherence; Lmod, leiomodin. (TIF) [file pbio.3000848.s001.tif]

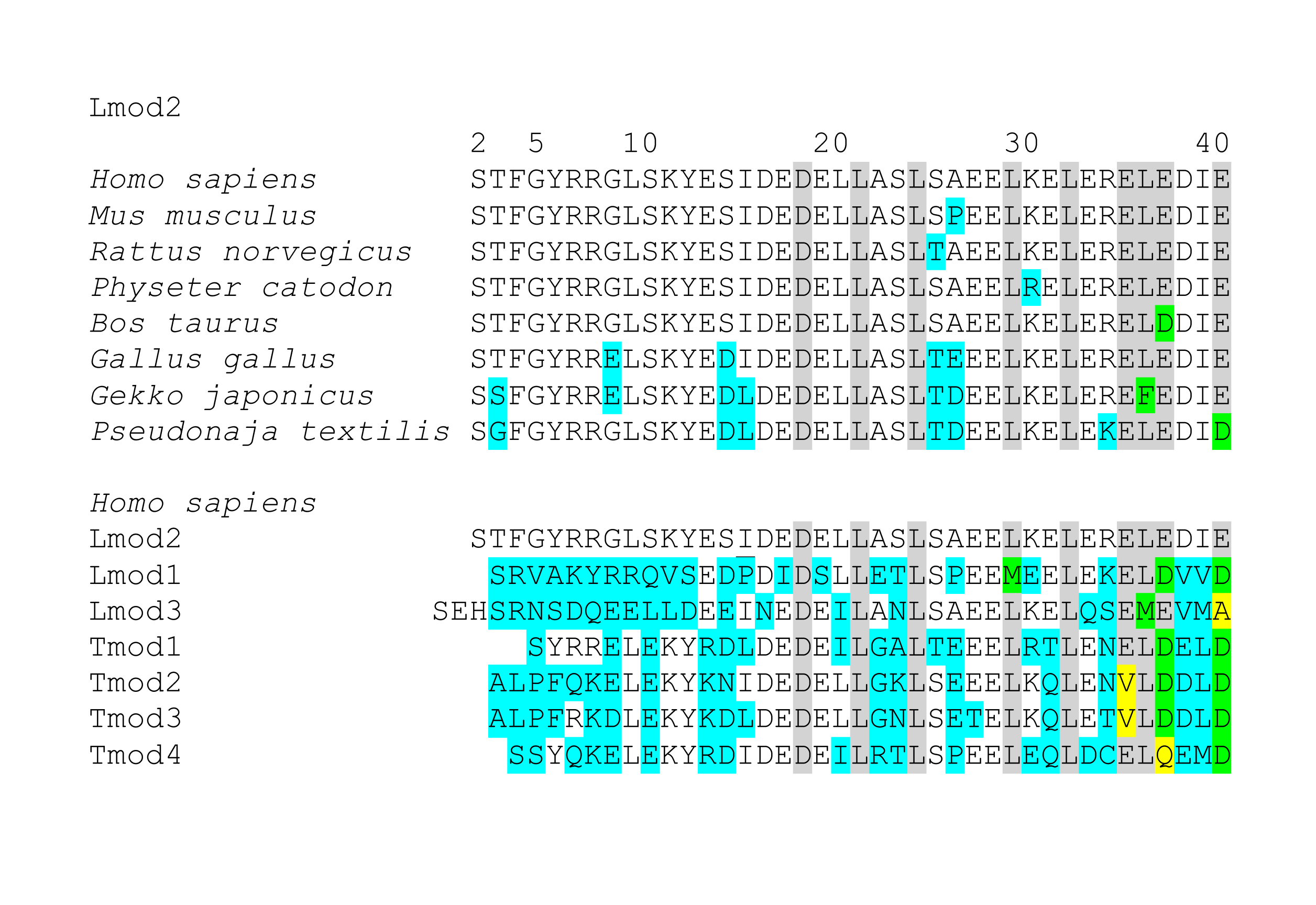

Supplement: S2 Fig — The sequence of human Lmod2 (from this study) is used as a reference in the comparison. Conserved residues forming the TpmBS1/tropomyosin binding interface are highlighted in gray. Conservative interfacial residue replacements of similar volume [80] and charge/hydrophobicity are highlighted in green. Nonconservative interfacial replacements are highlighted in yellow. Sequence replacements of residues that are not in direct contact with tropomyosin are highlighted in cyan. Lmod, leiomodin; Tmod, tropomodulin; TpmBS1, tropomyosin-binding site. (TIF) [file pbio.3000848.s002.tif]

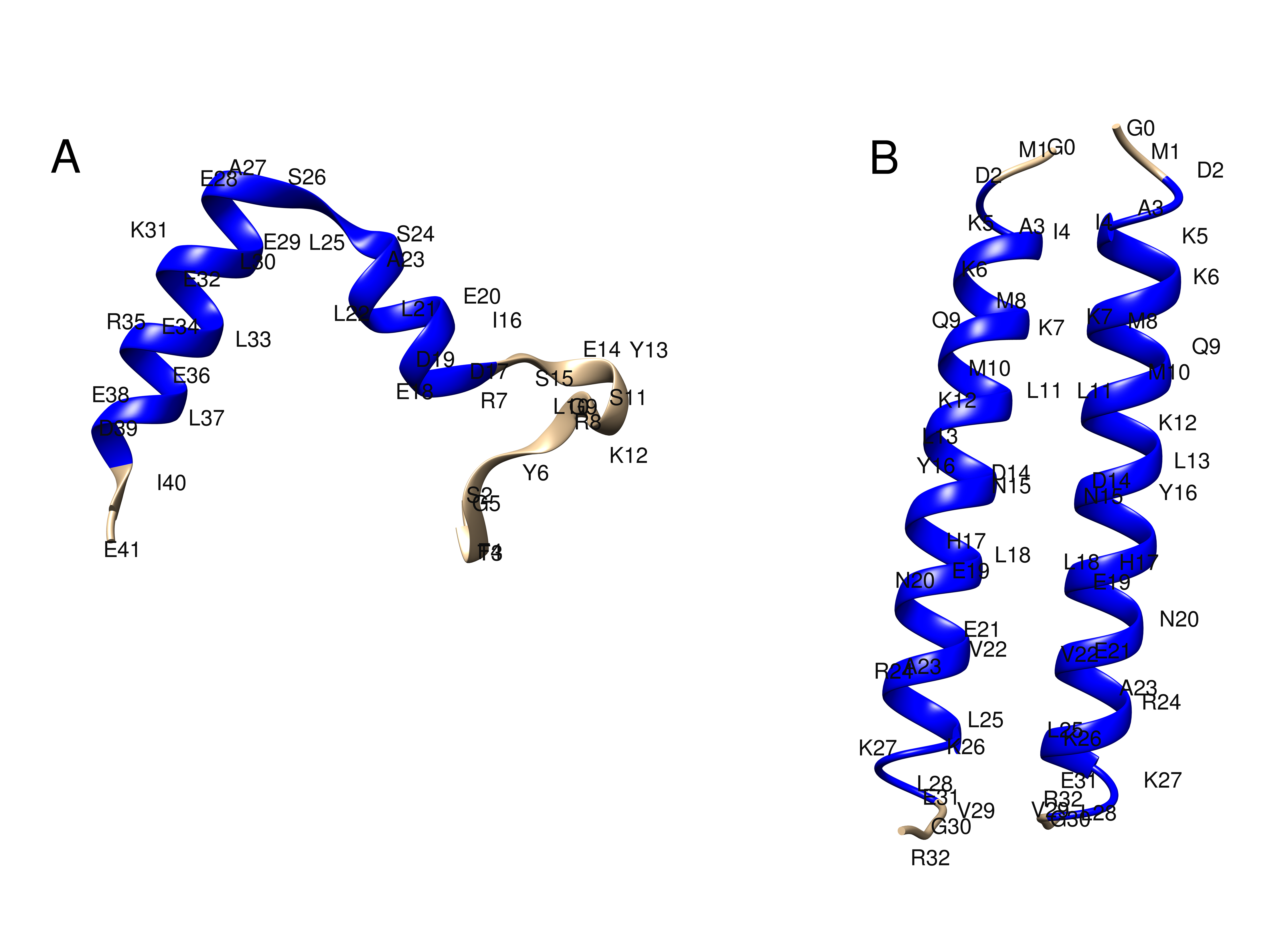

Supplement: S3 Fig — 3D conformations of the Lmod2s1 (A) and αTM1a1-14Zip (B) peptides in the Lmod2s1/αTM1a1-14Zip complex. (A) Backbone torsion angle values (φ,ψ) of Ile16-Ile40 amino acid residues were used to build a model of Lmod2s1. The well-structured region with RCI-S2 ≥ 0.7 (Asp17-Asp39) is shown in blue. (B) Backbone torsion angle values (φ,ψ) of Asp2-Gly30 amino acid residues were used to build a model of the coiled-coil αTM1a1-14Zip peptide. α-helices (Asp2-Leu28) are shown in blue. 3D, three-dimensional; Lmod, leiomodin. (TIF) [file pbio.3000848.s003.tif]

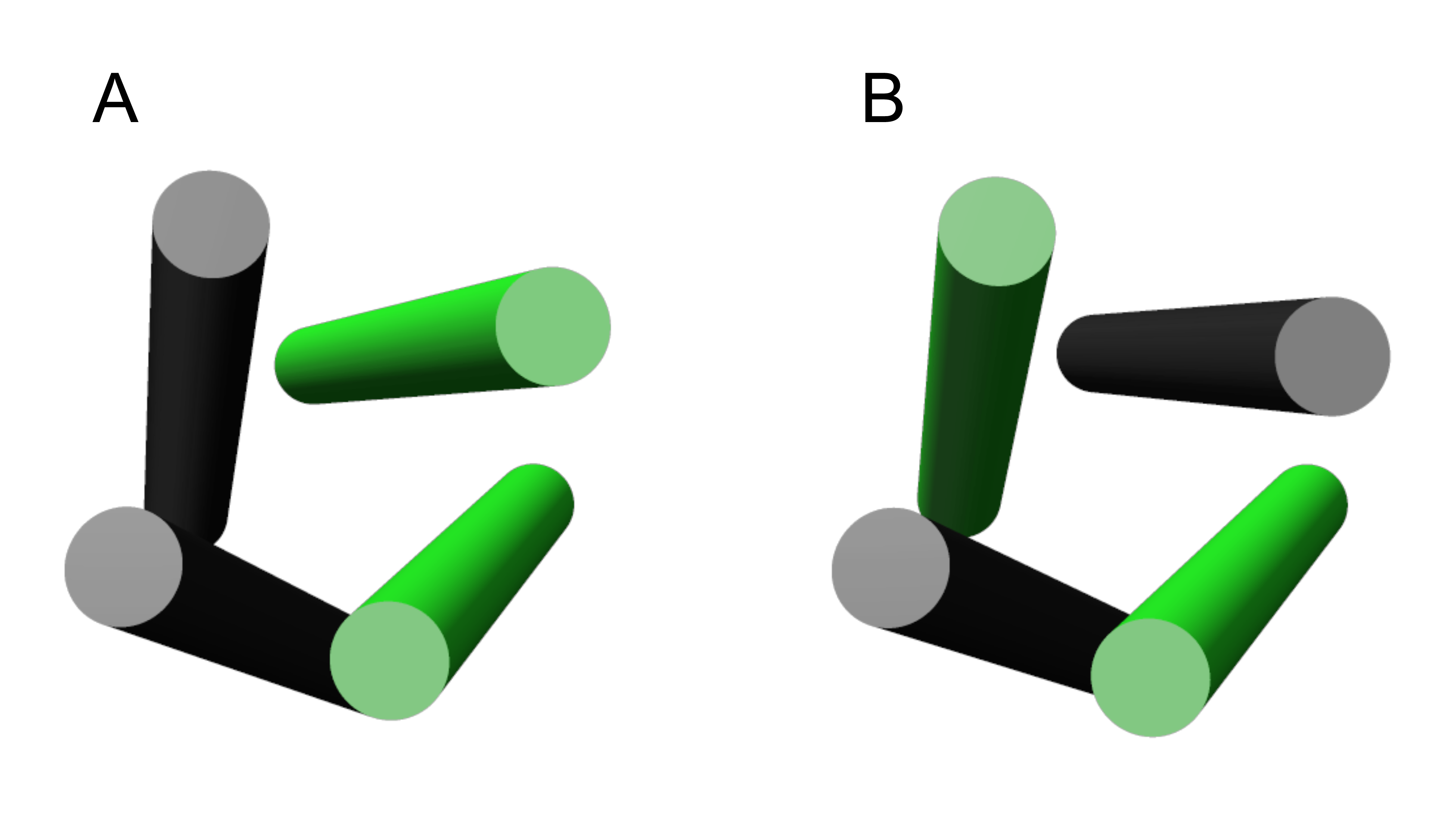

Supplement: S4 Fig — Lmod2s1 helices are shown in green, and αTM1a1-14Zip helices are shown in black. 1YO7 (ID PDB) 4-helix bundle was used as the topology template. (A) Side-by-side topology, and (B) crisscross topology. Lmod, leiomodin; MDS, molecular dynamics simulation; PDB, Protein Data Bank. (TIF) [file pbio.3000848.s004.tif]

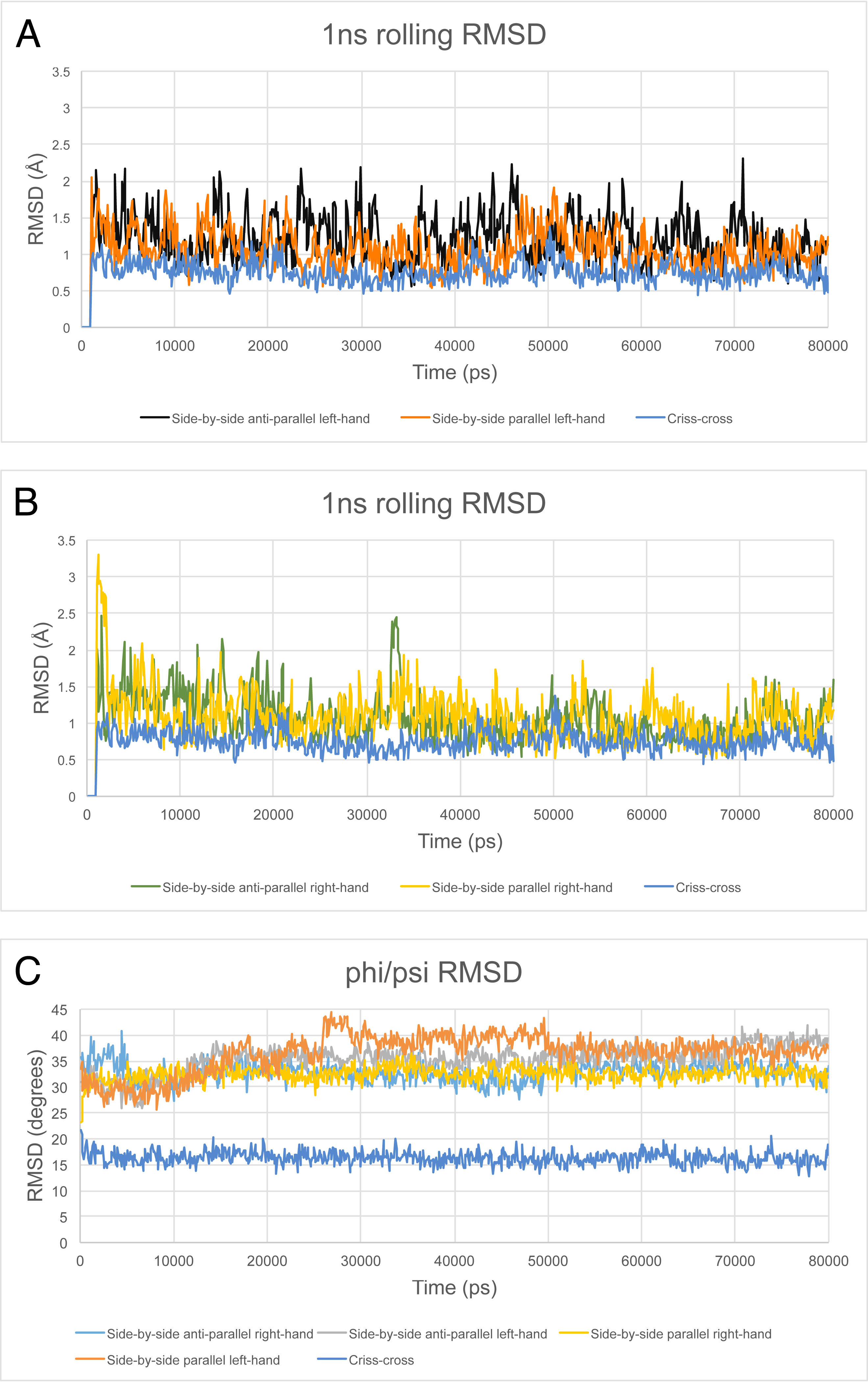

Supplement: S5 Fig — The rolling RMSD was calculated between two structures of the complex, one current and another one observed 1 ns before the current, as described in Materials and methods. RMSD values were averaged between two replicas. Four versions of side-by-side helix packing were tested, with two of them representing approximately parallel orientation of the C-terminal (residues Ala27-Glu38) Lmod2s1 helix with respect to the αTM1a1-14Zip, and the other two antiparallel. For each of the two orientations of the helix, one of two sides of Lmod2s1 can interact with the αTM1a1-14Zip. These sides were denoted as the left-hand side (when looking at Lmod2s1 in the direction from the N-terminal [residues Glu18-Ser24] to the C-terminal helices) and the right-hand side. (A) The rolling RMSD for left-hand side-by-side helical packing in comparison with the crisscross packing. The mean ± SD RMSDs (in Å) were equal to 0.76 ± 0.14 (crisscross), 1.06 ± 0.25 (parallel left-hand), and 1.22 ± 0.31 (antiparallel left-hand). (B) The rolling RMSD for right-hand side-by-side helical packing in comparison with the crisscross packing. The mean ± SD RMSDs (in Å) were equal to 1.09 ± 0.34 (parallel right-hand) and 1.08 ± 0.30 (antiparallel right-hand). (C) The (φ,ψ) RMSD between the current structure and the TALOS+ prediction. MDS, molecular dynamics simulation; RMSD, root-mean-square deviation. (TIF) [file pbio.3000848.s005.tif]

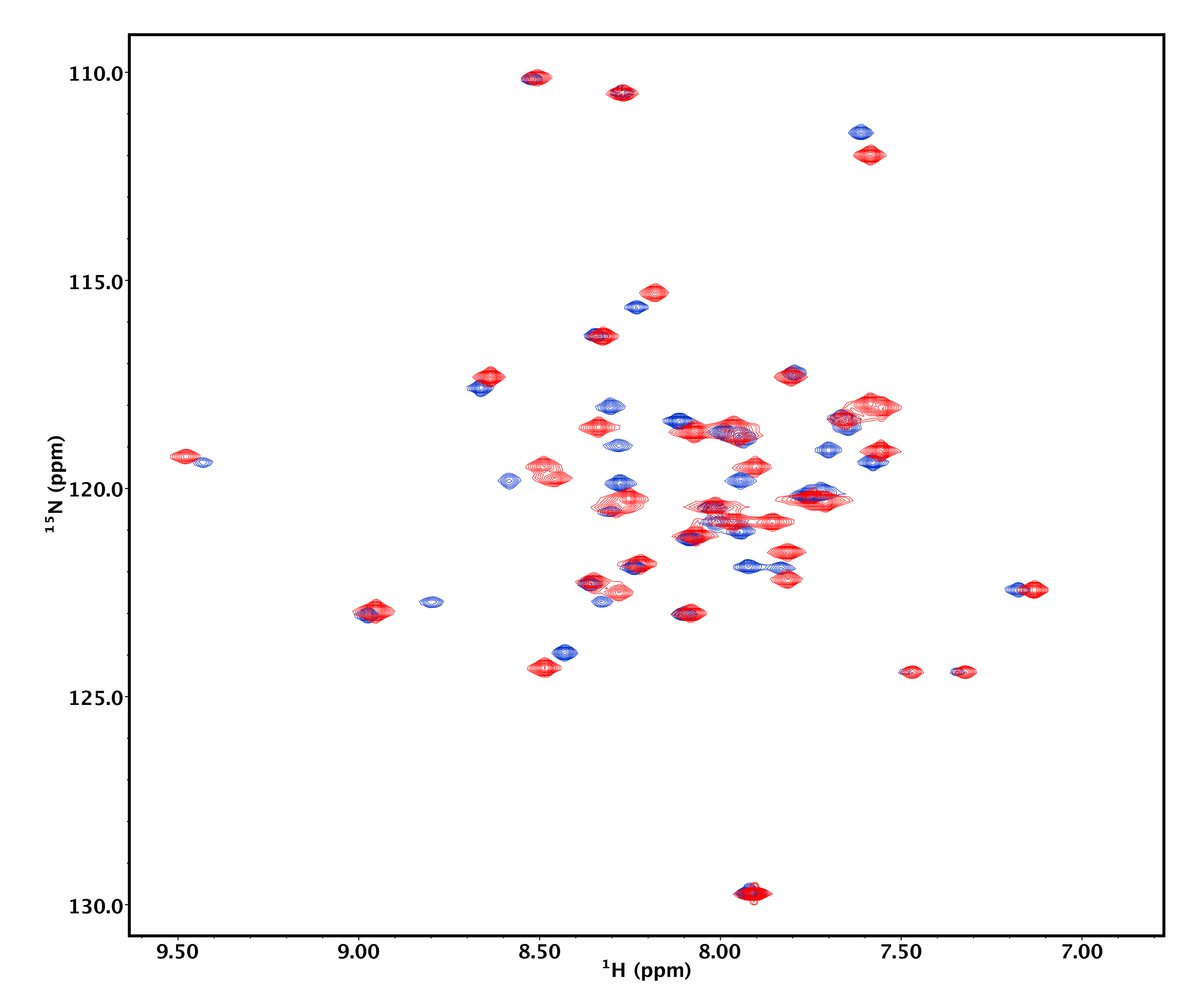

Supplement: S6 Fig — Spectra of approximately 0.3 mM 15N-Lmod2s1 in the presence of roughly 2× excess of the corresponding unlabeled tropomyosin coiled-coiled peptide were recorded on a Varian VNMRS 600 MHz spectrometer at 25°C. The general pattern of resonance peak distribution upon the substitution of αTM1a1-14Zip with Ac-αTM1a1-14Zip was preserved, confirming that both complexes have a similar structure. HSQC, heteronuclear single-quantum coherence. (TIF) [file pbio.3000848.s006.tif]

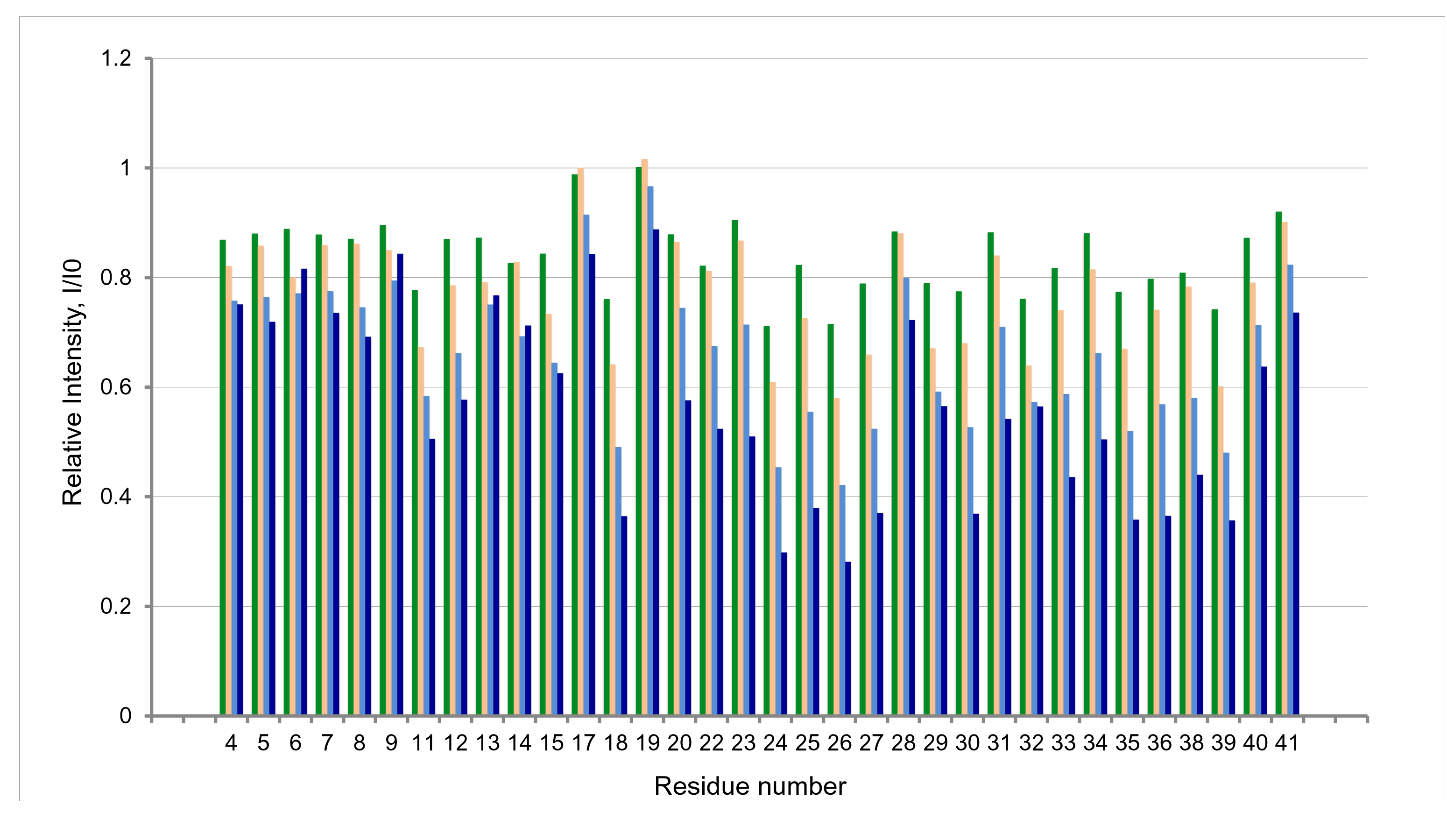

Supplement: S7 Fig — x-Axis shows the Lmod2s1 residue number. Peak intensities in the absence of αTM1a1-28Zip (I0) were considered equal to 1. Lmod, leiomodin. (TIF) [file pbio.3000848.s007.tif]

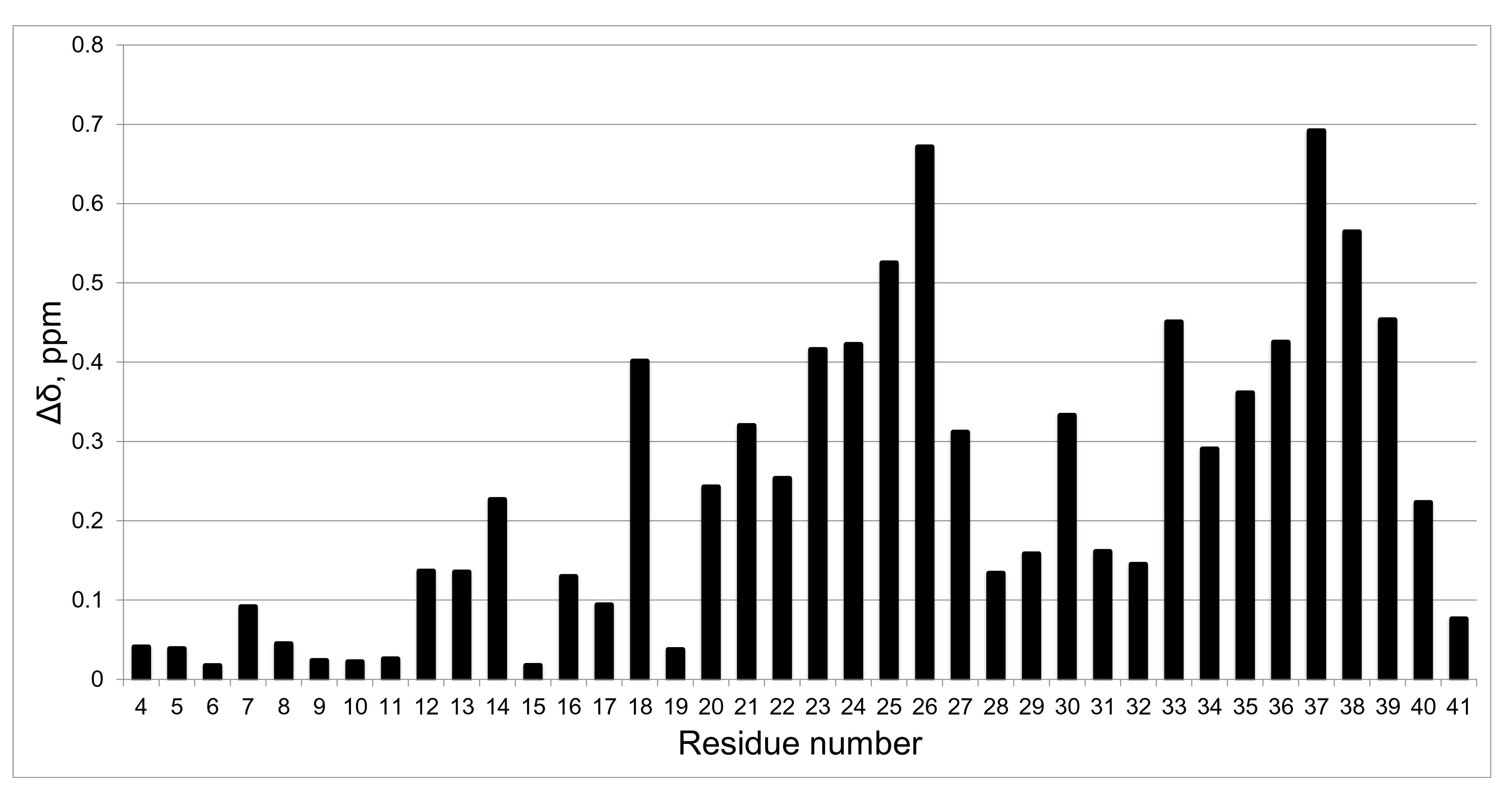

Supplement: S8 Fig — The compound chemical shift changes (S5 Data) were calculated as Δδ = [(ΔδH)2 + (ΔδN/6.5)2]1/2 [79]. Lmod, leiomodin. (TIF) [file pbio.3000848.s008.tif]

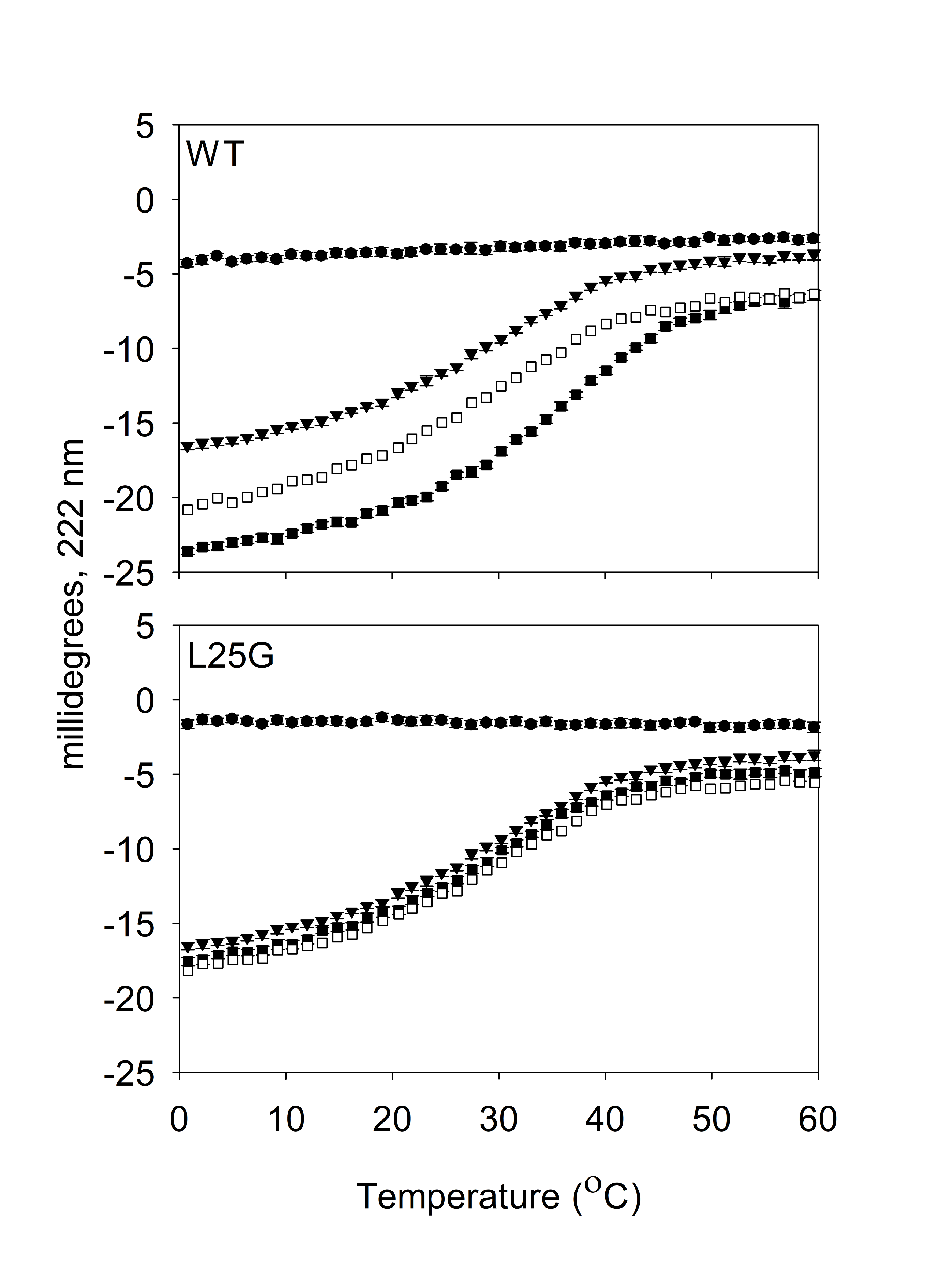

Supplement: S9 Fig — Peptide concentrations were 10 μM in 10 mM sodium phosphate buffer (pH 7.0) and 100 mM NaCl. The melting curve of the mixture of Lmod2s1 with αTM1a1-14Zip is different from the arithmetic sum of the individual melting curves of free Lmod2s1 and αTM1a1-14Zip, indicating that there is interaction between the two peptides. Melting temperature is higher for the mixture, which is consistent with a binding-induced increase in overall α-helical content in the complex [26, 27]. The melting curve of Lmod2s1[L25G] and αTM1a1-14Zip mixture is practically identical to the arithmetic sum of the individual curves of free Lmod2s1[L25G] and αTM1a1-14Zip, indicating no interaction under these conditions. ●, Lmod2s1; ▼, αTM1a1-14Zip; ■, Lmod2s1 and αTM1a1-14Zip mixture; □, arithmetic sum of melting curves for Lmod2s1 and αTM1a1-14Zip; Lmod, leiomodin. (TIF) [file pbio.3000848.s009.tif]

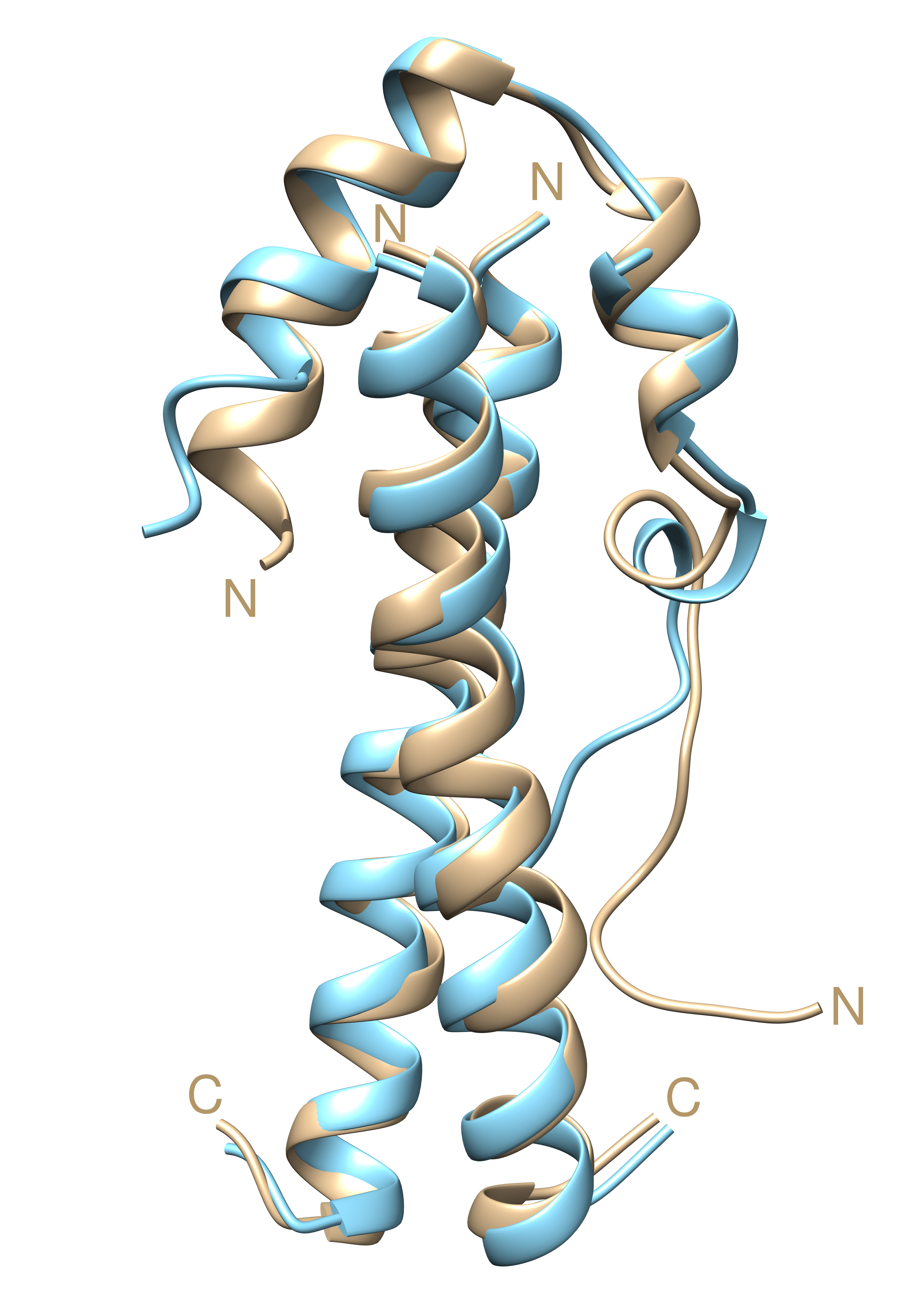

Supplement: S10 Fig — Lmod2s1 sequence in the Lmod2s1/αTM1a1-14Zip complex (Fig 1) was substituted with the homologous sequence from Tmod1 (Tmod1s1), and the Tmod1s1/αTM1a1-14Zip complex was subjected to a 400-ns MD simulation. A minimal-energy structure (achieved after about 317 ns) was used for comparison. Lmod, leiomodin; MD, molecular dynamics; Tmod, tropomodulin; TpmBS1, tropomyosin-binding site. (TIF) [file pbio.3000848.s010.tif]

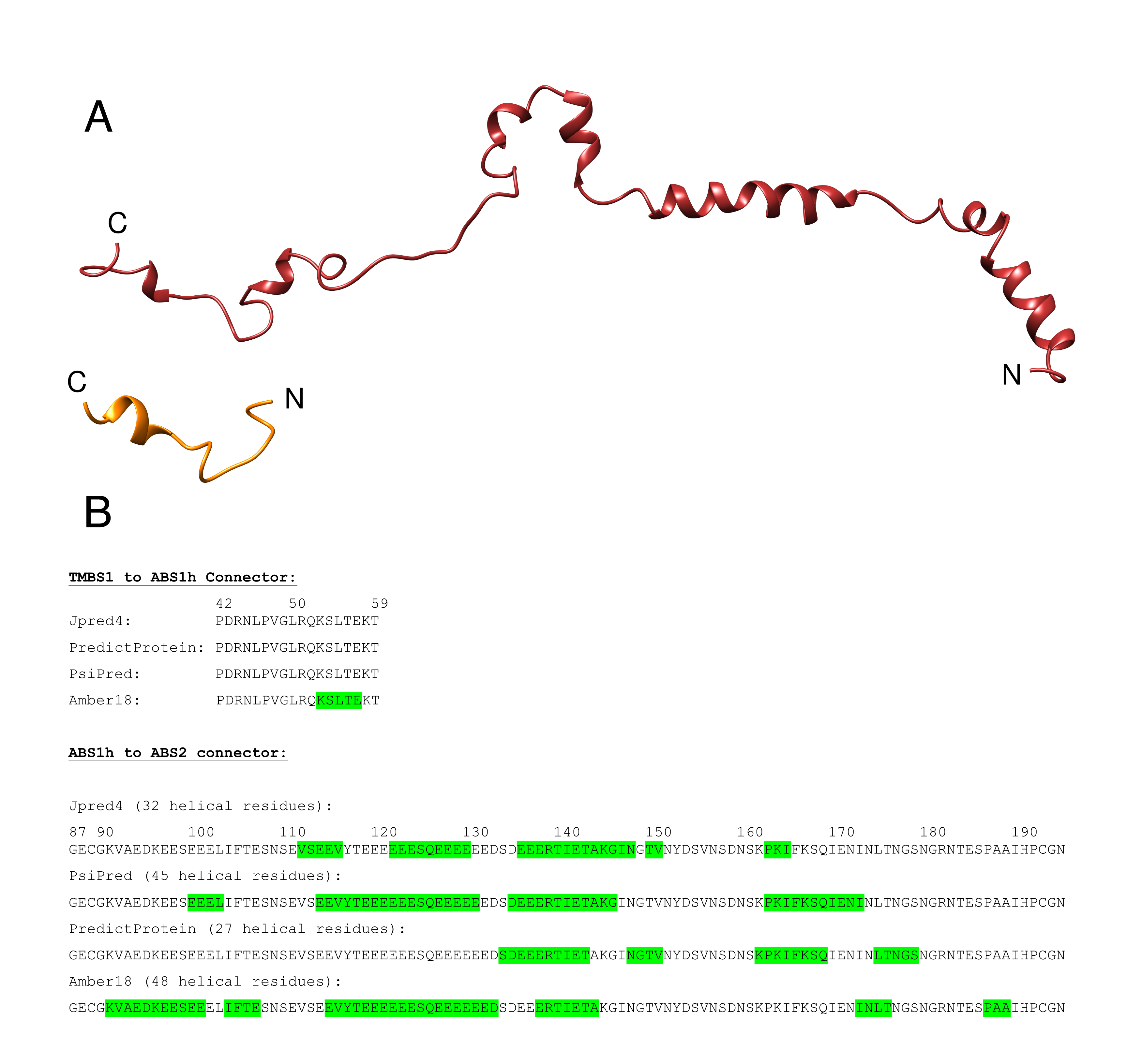

Supplement: S11 Fig — (A) Secondary structure elements in the linkers as predicted by Amber18. The fragment Pro42-Thr59 to connect TpmBS1 and ABS1h is colored in orange. The fragment Gly87-Asn195 to connect ABS1h and ABS2 is colored in brown. The secondary structure elements (α-helices, shown as coiled ribbons) were formed after 50 ns of MDS. Residues that did not form a regular secondary structure were considered disordered/flexible. The contour lengths of the linkers including the modeled α-helices were estimated as 59.5 Å (Pro42-Thr59) and 306 Å (Gly87-Asn195) and were sufficiently large to connect TpmBS1, ABS1h, and ABS2 at the pointed end without steric clashes. (B) Comparison of secondary structure predictions made by Amber18, Jpred4, PsiPred, and PredictProtein for linkers Pro42-Thr59 and Gly87-Asn195. Predicted α-helices are highlighted in green. 3D, three-dimensional; ABS, actin-binding site; Lmod, leiomodin; MDS, molecular dynamics simulation; TpmBS1, tropomyosin-binding site. (TIF) [file pbio.3000848.s011.tif]

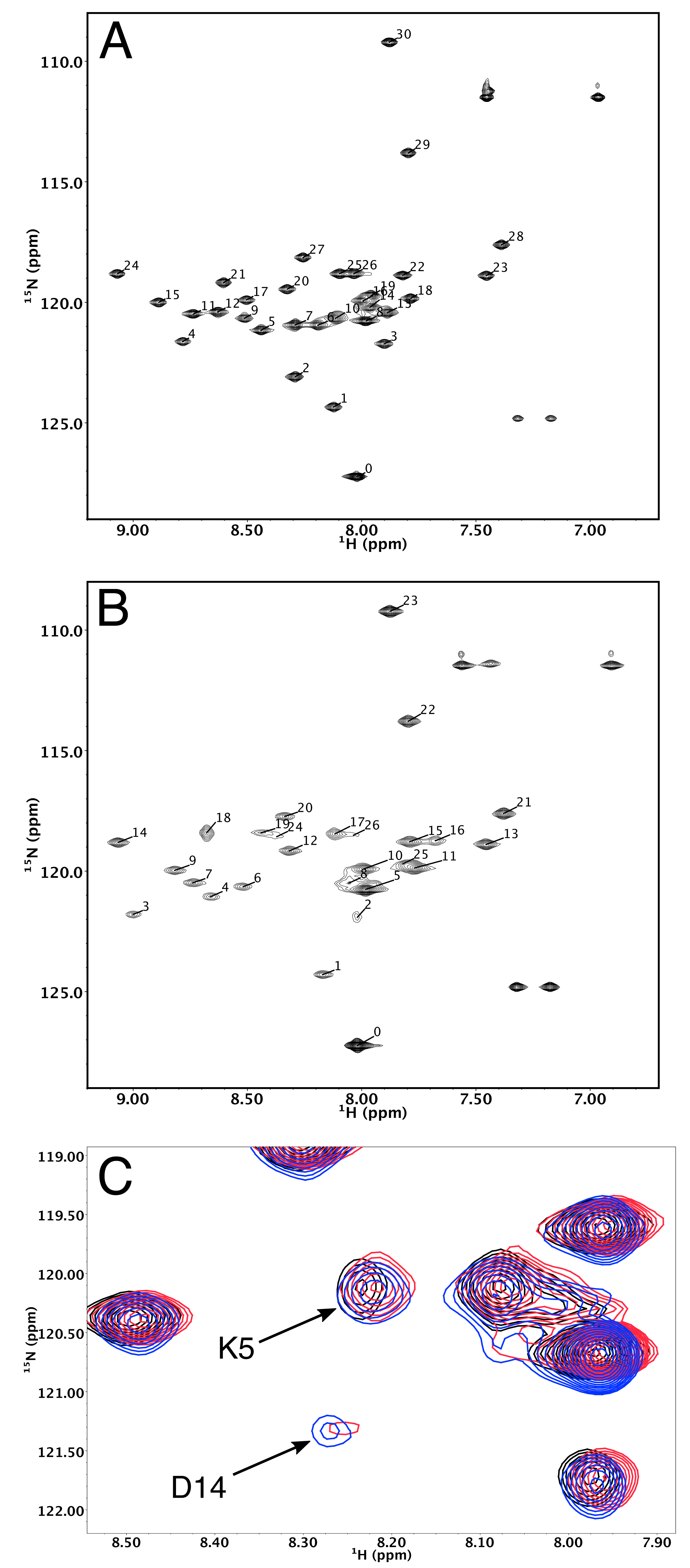

Supplement: S12 Fig — The spectra were recorded in 50 mM sodium phosphate buffer (pH 6.5), 10% D2O, 0.2 mM EDTA, 0.1% sodium azide, 2X Pierce EDTA-free protease inhibitor cocktail. (A) and (B) are the spectra of 15N/13C-labeled αTM1a1-14Zip (0.45 mM) in the absence and presence of the excess Lmod2s1 (1.5 molar ratio), respectively. Before the Lmod2s1 addition (spectrum A), 30 resolved cross-peaks corresponding to the backbone amides of αTM1a1-14Zip (MW ≈ 8.2 kDa) were detected. Upon the Lmod2s1 (MW ≈ 4.7 kDa) addition (spectrum B), the cross-peaks exhibited non-uniform broadening. As a result of the broadening, the peak intensities decreased, and the total number of the detectable resolved peaks reduced to 23. Additionally, some of the detectable peaks were disproportionally weak (see, e.g., peaks 2, 19, 24, and 26 in spectrum B). The non-uniform broadening suggested that the complex undergoes chemical exchange. The spectra (A) and (B) were recorded at 600 MHz, 10°C. (C) displays an overlay of 2D 15N-HSQC spectra regions of 15N/13C-labeled αTM1a1-14Zip in the presence of three different molar ratio of Lmod2s1: αTM1a1-14Zip, i.e., 1.5 (black), 2.4 (red), and 3 (blue). As the Lmod2s1 concentration increased, we observed an increase of some peak intensities (e.g., cross-peaks from K5 and D14 amides), confirming that the processes of association/dissociation of the complex contribute to the line broadening. The spectra (C) were recorded at 500 MHz, 25°C. 2D, two-dimensional; HSQC, heteronuclear single-quantum coherence; Lmod, leiomodin; MW, molecular mass. (TIF) [file pbio.3000848.s012.tif]

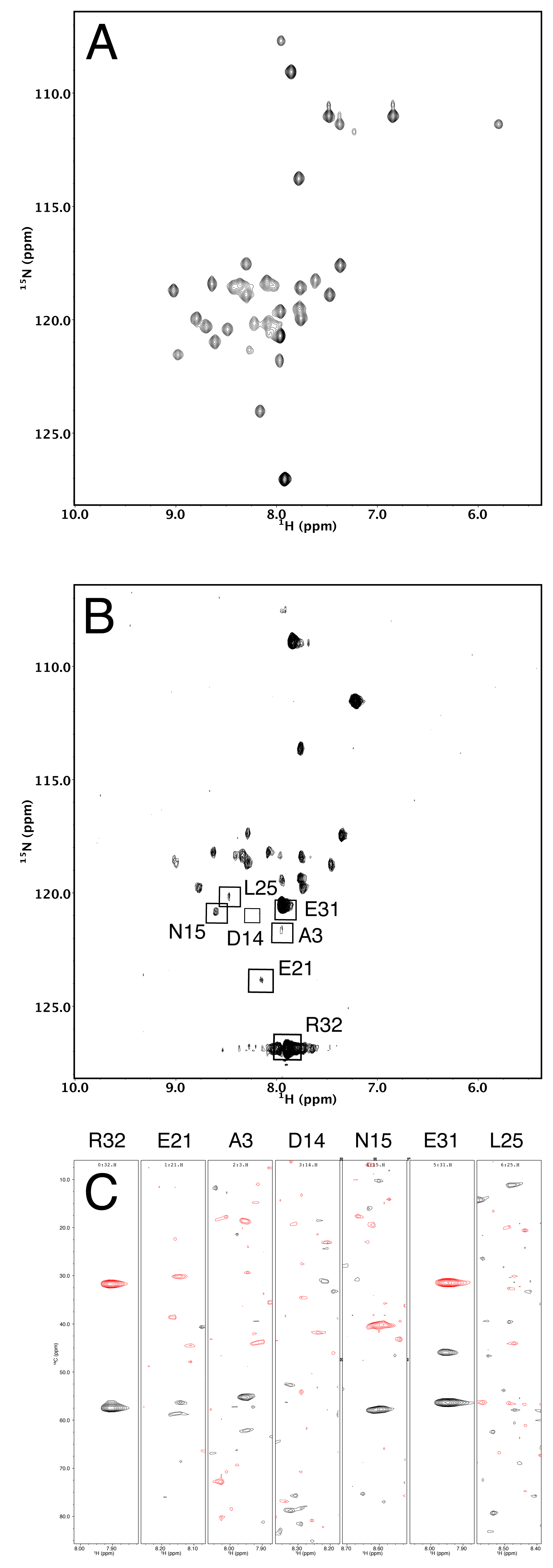

Supplement: S13 Fig — (A) A 2D 15N-HSQC spectrum of 15N/13C-labeled αTM1a1-14Zip (0.45 mM) in the presence of 1.35 mM Lmod2s1 ([Lmod2s1]:[αTM1a1-14Zip] = 3:1). (B) A 2D [15N, 1H]-plane projection of a 3D HNCACB spectrum recorded for the same sample as used in (A). To observe low-intensity peaks, the base contour level was lowered to slightly above the level of noise. Nevertheless, some of the cross-peaks from backbone amides seen in (A) are not present in (B), while many other peaks are weak. Boxed are peaks corresponding to NH strips in panel (C). (C) Strip view of the HNCACB spectrum. Shown in the panel are backbone amide NH strips corresponding to peaks boxed in (B). To observe low-intensity peaks, in strips with weak cross-peaks the base contour level was lowered to the level of noise. Positive and negative peaks are represented by black and red contours, respectively. In many amide NH strips, cross-peaks from Cα(i-1)/Cβ(i-1) of the preceding residue cannot be detected. Notwithstanding the line broadening, a combination of several 3D spectra enabled an almost complete assignment of backbone atoms and partial assignment of side-chain atoms in the complex (see the list of collected spectra in the Materials and Methods). The 2D 15N-HSQC and 3D HNCACB spectra were recorded in 50 mM sodium phosphate buffer (pH 6.5), 10% D2O, 0.2 mM EDTA, 0.1% sodium azide, 2X Pierce EDTA-free protease inhibitor cocktail, at 500 MHz, 25°C. 2D, two-dimensional; 3D, three-dimensional; HSQC, heteronuclear single-quantum coherence; Lmod, leiomodin. (TIF) [file pbio.3000848.s013.tif]

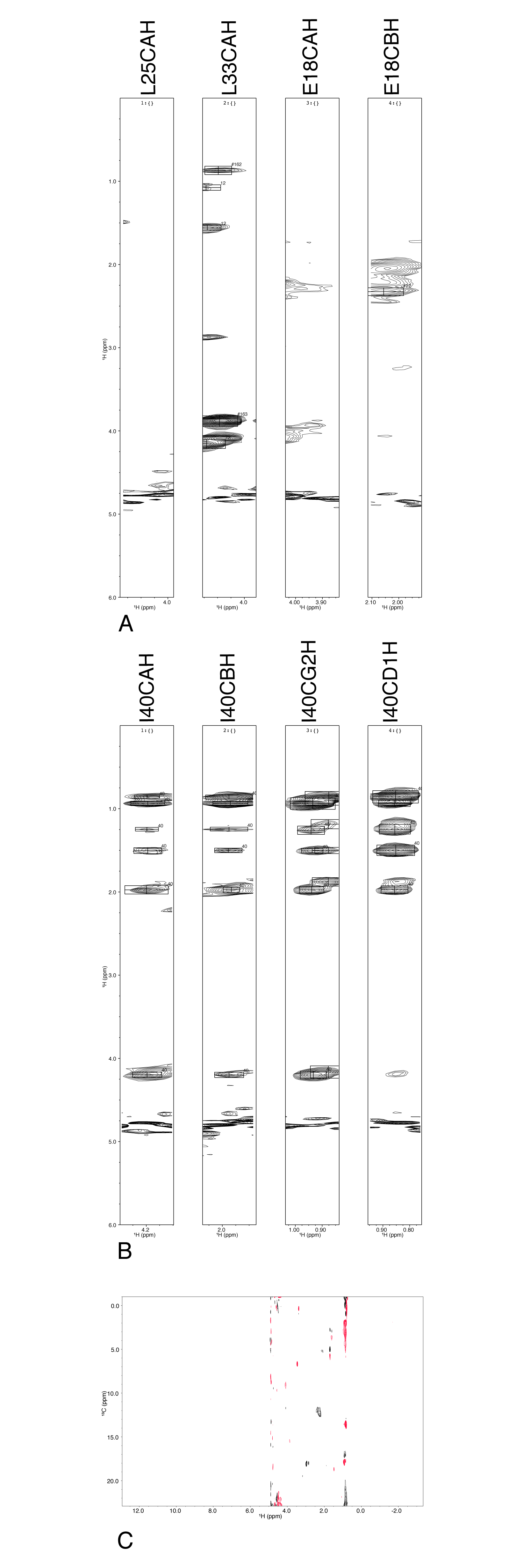

Supplement: S14 Fig — (A) Non-overlapped CH strips of Leu25 (CαH), Leu33 (CαH), Glu18 (CαH), Glu18 (CβH). All Leu25 and Leu33 cross-peaks and some E18 cross-peaks are broadened beyond detection. (B) Shown for comparison are non-overlapped CH strips of I40 (CαH, CβH, Cγ2H, Cδ1H) with sharp cross-peaks and clearly identifiable cross-peak patterns of an Ile side chain. (C) A CH projection of a 13C-filtered NOESY. Only a few cross-peaks are observed in the spectrum. Positive and negative peaks are represented by black and red contours, respectively. Lmod, leiomodin. (TIF) [file pbio.3000848.s014.tif]

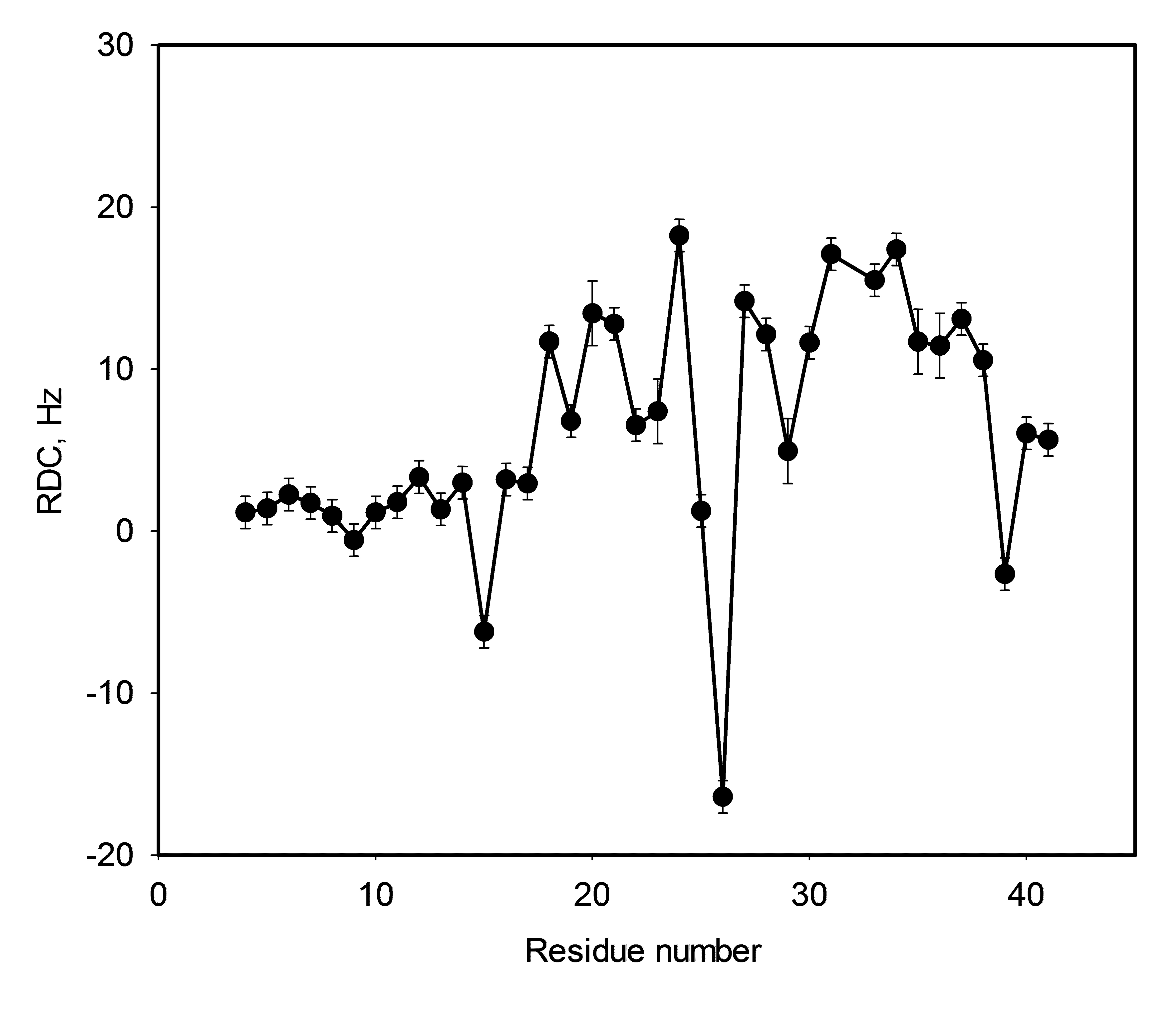

Supplement: S15 Fig — Uncertainties in RDC determination are shown as vertical bars. The dependence of RDC values on residue number for rigid Glu18-Ser24 and Ala27-Glu38 α-helices of Lmod2s1 follows oscillatory patterns as predicted for regular secondary structure elements (S7 Data) [81, 82]. The fitting of the observed RDCs to a pattern expected for an ideal α-helix gave each helix a set of parameters AI, AII, and Aυ, which depend on the orientation of the helices with respect to a coordinate system bound to the molecule [81]. AII and Aυ represent dominant sinusoid amplitude and an average RDC value, respectively, for an ideal α-helix [81], and they were found to be different for each of the two helices (AII~5.2 ± 0.2 Hz, Aυ = 11.0 ± 0.1 Hz for Glu18-Ser24 and AII = 2.7 ± 0.2 Hz, Aυ = 12.2 ± 0.1 Hz for Ala27-Glu38). Specifically, the amplitude AII was larger for helix Glu18-Ser24, and Aυ was slightly larger for residues Ala27-Glu38. According to the theoretical expressions for AII and Aυ [81], the helices are angled with respect to each other in the complex, in agreement with the result obtained by TALOS+. Lmod, leiomodin; RDC, residual dipolar coupling. (TIF) [file pbio.3000848.s015.tif]

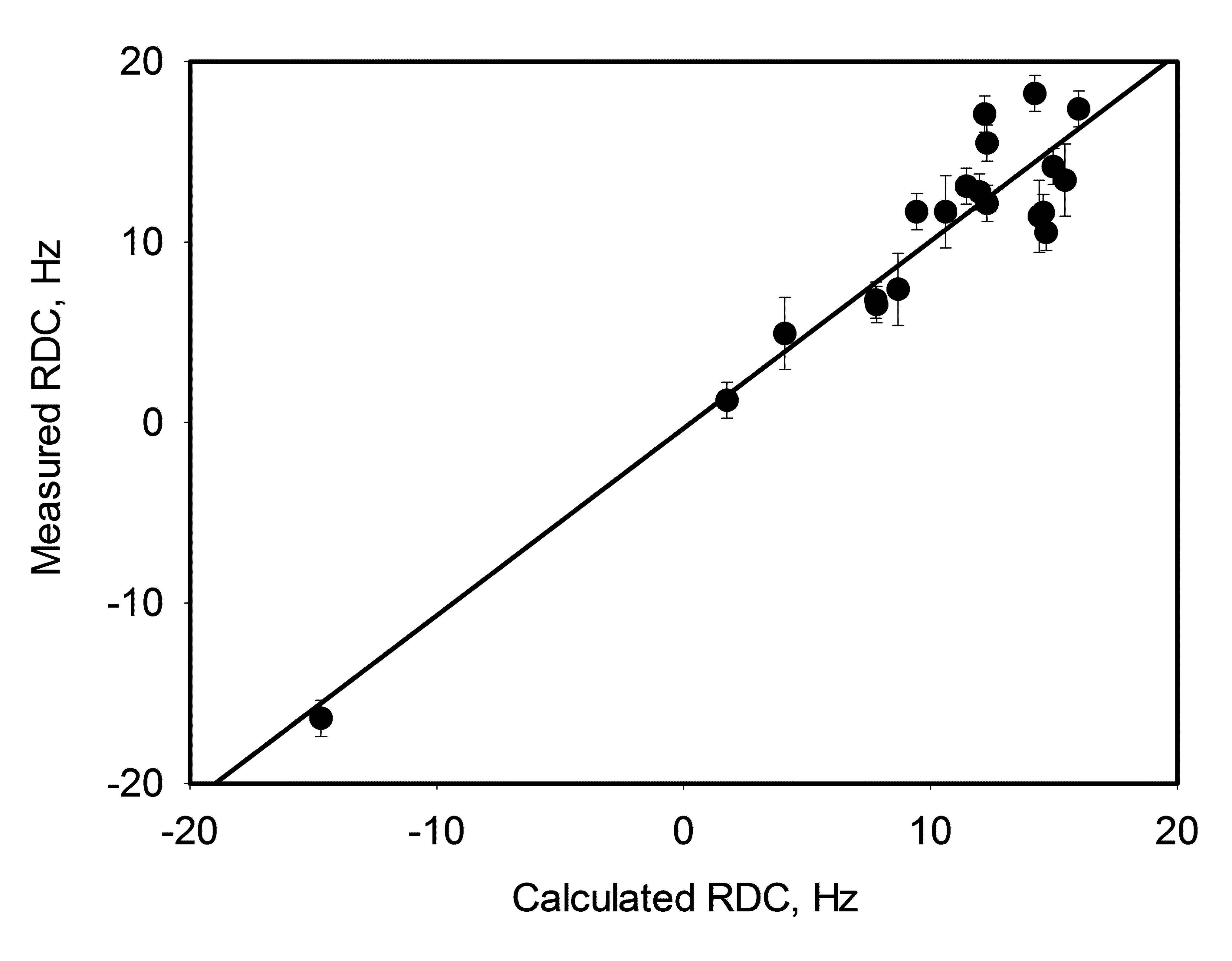

Supplement: S16 Fig — Vertical bars show estimated uncertainties in RDC values. RDC, residual dipolar coupling. (TIF) [file pbio.3000848.s016.tif]
