# Supplementary material for: Leiomodin creates a leaky cap at the pointed end of actin-thin filaments
Source: PLoS Biol. 2020 Sep 8;18(9):e3000848. doi: 10.1371/journal.pbio.3000848 (PMC7500696; doi:10.1371/journal.pbio.3000848)
Supplement: Attachment — Submitted filename: Response_to_reviewers_Tolkatchev.pdf [file pbio.3000848.s033.pdf]

Response to reviewers.

We thank the reviewers for their critiques and valuable comments, which helped to improve our manuscript. All pages and line numbers are given for the manuscript version with tracked changes.

Reviewer #1:
Q1: *Figure 7: It would be easier to interpret this figure, if actin monomers would be presented in two different shades of color? ABS2 is shown in magenta not purple? Its structure is modelled from the tropomodulin structure? Clearly state this in legend and text.*

A:

The actin molecule A is now shown in tan, and actin molecule B is shown in gray. The color of ABS2 now reads as "magenta" in the figure legend. The known structures that were used for building the model are now explicitly indicated in the legend to Figure 7 (pp 35-36, lines 1126, 1131) and the main text (p14, lines 392-395).

Q2: *Figure 8: turquoise new actin attaches to actin A not B as stated?*

A:

The error was corrected (B changed to A) (p.36, line 1143)

Reviewer #2:
Q3: *The authors mention that the assignment is incomplete due to resonance overlap. However, a broad range of triple resonance experiments were measured yet one of the most important ones that helps in this case - of the type of HN(CO)CCH or HNCCH - were not recorded. Given the size of the complex and the relatively high concentration this should be feasible. These experiments provide all side chain proton and carbon resonances at the resolution of the HSQC which is excellent. A more complete assignment will also be useful in better ways of model building.*

A:

The experiments proposed by the reviewer would be perfectly relevant for a "NMR-friendly", high-affinity (with K_d in nM range) protein complex. Unfortunately, this is not the case for the molecular complex we studied. The main reason for incomplete side chain assignment is not the signal overlap issues, but line broadening, for some peaks beyond detection, as a result of chemical exchange.

The complex undergoes association/dissociation exchange process, which contributes markedly to nuclear magnetic relaxation and limits the amount of obtainable NMR spectral information. In each NMR sample one of the two peptides in the Lmod2s1/Tpm1.1 peptide complex was labeled while the other one was unlabeled. At ~1.5-fold stoichiometric excess of the unlabeled peptide component, we estimated the apparent rotational correlation time τ_c of the $^{15}N/^{13}C$-αTM1a$_{1-14}$Zip/Lmod2s1 complex to be roughly 17 ns. This exceeded more than two-fold a typical τ_c (~7.2 ns) for a rigid and spherical protein of this size (~12 kDa). Since we found that an increase of the concentration of the unlabeled peptide led to narrowing of the resonance lines, we concluded that

it was the chemical exchange between bound and traces of unbound labeled peptides (~1% population for *Kd* in low µM range (Colpan et al (2016) Biochim Biophys Acta. 1864(5): 523–530)) that was causing the elevated T2 relaxation rate. This observation is not surprising because on-rate kinetic constants for protein association are typically in the range of ~10^5-10^8 $M^{-1}s^{-1}$ (Schreiber et al (2009) Chem Rev. 109(3): 839–860), potentially making the exchange rate (k_{ex}=k_{off}+k_{on}×[unbound unlabeled peptide]≈ k_{on}×([total unlabeled]-[total labeled])~10^2-10^5 s^{-1}) for the complex close to intermediate on the chemical shift NMR time scale. Although some line narrowing could be achieved by the addition of the unlabeled peptide, we can only increase its concentration to approximately three-fold stoichiometric excess. If more unlabeled peptide was added, the lines would broaden (probably as a consequence of aggregation) and the complex would precipitate. Therefore, our ability to reduce the rate of T2 relaxation in the sample is limited by the solubility of the peptide components.

Resonance overlap exacerbated the problems in chemical shift assignment, but even if it did not exist, line broadening would still hamper complete spectral characterization of the side chains in the complex. Since resonance peaks for a number of residues were broadened beyond detection in ^{13}C-correlated 3D spectra, the combination of 3D spectra that we recorded was enough to reliably produce a near-complete assignment of backbone atoms but only partial assignment of side-chain atoms. In fact, some of the spectra we collected during the study were not mentioned or included in the manuscript, because line broadening rendered them practically unusable. For example, in the attempt to identify interchain contacts we collected 3D X-filtered NOESY spectra, but almost all cross peaks broadened out. Therefore, for this particular system it is very doubtful that new spectra will provide new reliable information that would fundamentally change the extent of chemical shift assignment and reduce the need for modeling.

To more clearly and explicitly communicate the limitations that were imposed on us by the association/dissociation of the Lmod2s1/αTM1a_{1-14}Zip complex, we have now introduced in the manuscript new or reformulated paragraphs and statements (p7, lines 179-181; pp19-20, lines 570-584; p20, lines 596-603).

Notwithstanding the difficulties with side chain assignments, chemical shift-based structure determination algorithms are validated by many members of protein NMR community; they are commonly used and widely accepted (reviewed in, e.g., Berjanski & Wishart (2017) Biochim Biophys Acta Proteins Proteom. 1865(11 Pt B):1564-1576; Nerli et al (2018) Prog Nucl Magn Reson Spectrosc. 106-107: 1–25). Importantly, experimental RDC values were not included as restraints in structure calculations but were only used for structure validation. In addition, we validated the specific use of chemical shift-based modeling applied to our complex by comparing the TALOS "predicted" structure of the coiled-coil tropomyosin component of the studied complex and its known NMR structure. The comparison is now included on pp21-22, lines 627-641:

Q4: *The authors indirectly create order parameters from chemical shifts. However, it would be much better to explicitly measure 15N relaxation data (heteronuclear NOE, T1, T2) which would give precise dynamics information residue by residue. Such data could also be used in the validation of the structure by considering anisotropic rotational diffusion of the complex.*

A:

We did not attempt direct measurements of relaxation parameters because of uncertain contributions of association/dissociation exchange to T2 values. We observed that ^{15}N-HSQC line widths were dependent on the concentration of the unlabeled peptide, meaning that chemical exchange between bound and traces of unbound labeled peptides contributed significantly to the T2 relaxation (see the answer to the previous question for more details). Considering that extracted from NMR data exchange parameters depend on the presumed mechanism of association/dissociation (Tolkatchev et al (2003) J. Am. Chem. Soc., 125 (41), pp 12432–12442), it remains unclear if direct measurements will produce more accurate information and provide more insight in the internal motions in the complex than values extracted from chemical shifts.

Since the order parameters that we obtained from TALOS are theoretical estimates, we only use them for qualitative assessment of the location of more rigid and more flexible regions. We did not base our assessment on the order parameters alone. We also used experimental data, or, more specifically, the change in chemical shifts of Lmod2s1 upon binding and RDC values, to support this assessment.

Q5: *It is most unfortunate the RDS could only be measured for one of the two peptides in the complex. Oriented media made from lipids or organic solvents are often challenging for certain proteins and it is not surprising that they caused problems in this case. Alternatives such as purple membrane or pf1 phage which are much more inert should be considered.*

A:

Since production of labeled αTM1a$_{1-14}$Zip is a low-yield, lengthy multi-step protocol (mainly due to the propensity of recombinant enterokinase (used in the purification protocol) to non-specific proteolysis), we carefully considered oriented media that can be used for our system. The complex is not long-lived and Lmod2s1 readily separates from the tropomyosin peptide αTM1a$_{1-14}$Zip when subjected to favorable for such separation conditions (e.g. an equilibrium shift), and therefore we eventually chose to use the neutral medium to perform RDC experiments. Our reasoning was that any charged surface would compete for binding with either Lmod2s1 (which is negatively charged at neutral pH due to high abundance of Glu and Asp residues) or αTM1a$_{1-14}$Zip (which is positively charged at neutral pH due to high abundance of Lys residues).

Tropomyosin peptide represents a coiled coil, which is very stable and does not change when it forms a complex. Therefore, the only important question for building the model that was left unanswered - when we found that we can only measure RDC values for Lmod2s1 - was not the structure of αTM1a$_{1-14}$Zip itself, but the mutual orientation of the tropomyosin peptide and Lmod2s1. We chose to answer this question by analyzing spectral effects of substitutions in tropomyosin peptides.

Q6: *In addition to using the RDCs there should be more precise measures of the consistency of the structure analogous to resolution in X-ray or RMSD in traditional NMR based structures. This will allow the reader a better assessment of the robustness of the structure.*

A:

Ten replica structures of the complex were generated by independent MDS production runs. For backbone N, Cα and C' atoms of well-defined regions (Asp17-Asp39 (Lmod2s1) and Asp2-

Leu28 (αTM1a$_{1-14}$Zip)) in the ten structures we calculated global pairwise RMSD (1.76±0.07 Å, mean±SEM). We also attached to Supporting information Table S3 detailing the pairwise RMSD statistics. This information is now included in the Materials and Methods on p24, lines 717-720 of the manuscript and in a newly provided supporting Table S3 (p.18, lines 179-184 of Supporting Information).

Q7: *The only use that the authors make from their assignments in structure calculation is the creation of dihedral angle constraints in TALOS+. It would be useful to consider alternative approaches that allow a much more comprehensive use of full assignments such as that provided by Rosetta-CS which is able to use the entire assignment and not only the backbone atoms and thus offers a much better representation of the experimental data. Additionally, by creating a structural model from protein fragments it makes for a less biased approach for model building.*

A:

To the best of our knowledge CS-Rosetta does not support calculations of polypeptide multimers unless they are symmetric. We contacted Dr. Wedell who is a Systems Programmer at the CS-Rosetta server at BioMagResBank and he confirmed that they only support continuous polypeptide chains and that he is not aware of the possibility to use CS-Rosetta for non-continuous polypeptide chains.

Reviewer #3:

Q8: *more details should be provided for the MD simulations performed on Lmod2s1/αTM1a1-14Zip. In particular:*
- shape of the box
- cutoff values used for non bonded interactions
- units of the force constants used for the dihedral restraints
- type of barostat with coupling constant

A:

Lines 664-675 (pp22-23) in Material and Methods were changed to introduce the requested information.

Q9: *MD simulations were performed also on the linkers connecting the Lmod2 binding sites, but no methodological details were provided. The Authors need to indicate the simulation parameters and the protocol followed for these systems. Also, it would be useful to know if the secondary structure from MD is consistent with secondary structure predictions (give for example by PSIPRED or analogous bioinformatics software). Additionally, it is unclear where these linkers are in Figure 7.*

A:

It is important to emphasize that our primary reason for the prediction of secondary structure elements in the linkers was to estimate their contour length and evaluate if they can connect TmpBS1 with ABS1h (called ABS1 in the first submission, more information on the reason for the nomenclature change is below) and ABS1h with ABS2. We were interested if the linkers can

work without the need to create additional strain by destroying putative secondary structure elements, rather than find the “true” conformation of the linkers, for which experimental data are not yet available. To make this clearer, we have revised lines 413-415 (p14).

To indicate the simulation parameters for the linkers we have now inserted in the Materials and Methods a paragraph on lines 733-737 (p25).

To demonstrate the comparison between the secondary structures obtained from MD and from secondary structure predictors we have added to Fig. S11 (former S10) predicted helical regions and introduced new pertinent information on p15 (lines 420-425) and p. 26 (lines 775-777) of the manuscript.

We can only model the structure of the part of Lmod2 ABS1 that is homologous to the corresponding part of actin-associated ABS1 in Tmod, for which the coordinates are known. This homologous region corresponds to residues Pro60-Leu86 in Lmod2. To make this part distinct from the putative full-size Lmod2 ABS1, we now call it in the manuscript as ABS1h (called ABS1 in the first submission). This also clarifies the definition of linkers. The linker connecting TmpBS1 with ABS1h consists of residues Pro42-Thr59 and the linker connecting ABS1h with ABS2 consists of residues Gly87-Asn195. We have now changed lines 396-409 on p14 to introduce this change of nomenclature.

To provide clarity on where these linkers are in Fig. 7, we changed the legend of Fig. 7 (lines 1140-1146 of pp40-41), which now states explicitly which residues constitute ABS1h and which constitute linkers connecting TpmBS1 with ABS1h or ABS1h with ABS2.

Q10: *More quantitative data should be provided for:*
- the preliminary runs on the alternative packing of the helices, showing the instability of the side-by-side and stability of the criss-cross arrangements in all the replicas.
- the results of the Simulated Annealing simulations: it is said that "The annealed structures were very similar", but this needs to be shown (for example with a superimposition of the structures or calculation of RMSD values).

A:

- *on the instability of alternative packing of the helices:* To characterize quantitatively the relative stability and fitness to experimental data of side-by-side and crisscross packing, we followed the evolution of two metrics along each trajectory. The first metric was “rolling RMSD” calculated between the current structure and the structure observed 1 ns before the current. It provided information on the nanosecond-scale intrinsic backbone motions in the structure. The second metric was a RMSD of backbone dihedral (φ,ψ) angles calculated between the current structure and the TALOS+ predicted values. The (φ,ψ) RMSD is a measure of how well the structure fits the experimental NMR data. Smaller (φ,ψ) RMSD meant smaller dihedral angle NMR constraint violations. Since αTM1a$_{1-14}$Zip forms a stable coiled coil and remained little changed in most simulations, we focused on Lmod2s1 to test the behavior of the metrics. The quantitative data is now shown in Fig. S5. The Fig. S5 demonstrates that structures representing the side-by-side topology had larger intrinsic backbone motions (manifested as larger mean rolling RMSD and larger standard deviation of rolling RMSD for each trajectory).

Importantly, the RMSDs of backbone dihedral (φ,ψ) angle for side-side structures were ~2.5-fold of those for crisscross helical packing, implying that the crisscross model fits the experimental NMR data much better. All this new information is now reflected on pp8-9 (lines 228-239 of the main text), pp24-25 (lines 721-732 of the Materials and Methods) and the legend to the new Fig. S5 (supporting information).

We also updated supplementary videos to videos S1-S5 to represent the runs described in the newly introduced paragraphs.

- *on the results of the Simulated Annealing simulations:* We introduced on pp23-24, lines 690-711 a paragraph, which A) provides results of quantitative analysis for annealed replicas, and B) clarifies what was similar between the structures; also, a Table S2 with respective RMSD values was added to supporting information (p17, lines 167-178).

Q11: *at page 13, lines 355-356: "confirming that the N-terminus of tropomyosin cannot accommodate a simultaneous binding of both Tmod1 and Lmod2" is too strong considering how this result was obtained (simple replacement of the sequence followed by a relatively short MD simulation). The statement should be revised to highlight that this is a model.*

A:

We have changed the statement to

"This modeling result provides evidence in favor of our proposition that the N-terminus of tropomyosin cannot accommodate a simultaneous binding of both Tmod1 and Lmod2." (p13, lines 374-375):

Q12: *the final structures of the models should be made available as supplementary data.*

A:

A cluster of ten structures of the Lmod2s1/αTM1a$_{1-14}$Zip complex was submitted to RCSB PDB (pdb# 6UT2) and the coordinates will be released once the manuscript is accepted for publication. Pairwise RMSD between models in the cluster were calculated using coordinates of superimposed backbone atoms N, Cα, and C' of well-defined regions in the molecular complex (Asp17-Asp39 in Lmod2s1 and Asp2-Leu28 in αTM1a$_{1-14}$Zip). The RMSD sample mean and SEM were calculated at 1.76 Å and 0.07 Å, respectively. The RMSD information is now included on p24, lines 717-720 of the manuscript and presented in the supporting Table S3 (p.18, lines 179-184 of Supporting Information).

We believe that it is premature to publish the pdb coordinates of our model of Lmod2 pointed end assembly. Neither the structure of the linkers connecting TmpBS1, ABS1 and ABS2 nor the linkers' possible interaction interface with actin is currently available. Publishing such pdb might mislead a reader into believing that coordinates of atoms in the linkers are accurate and are based on experimental structural data. Our only goal in the attempt to link TmpBS1, ABS1 and ABS2 was to make sure that linkers including secondary structure elements predicted by MDS are long enough to provide the connections and not cause steric clashes with the filament. By introducing

the linkers into the assembly we were mainly testing the structure-based feasibility of our proposed mechanism, rather than trying to establish true coordinates of atoms in the linkers. On the other hand, if the reviewers find it useful, supplying the pdb coordinates for a model of the Lmod assembly at the pointed end that does not include the linkers can certainly be done.

Additionally, to provide readers with means to check our conclusions we will deposit trajectories and input files for the Lmod2s1/αTM1a$_{1\text{-}14}$Zip complex and the linkers to the PLOS Biology-recommended data sharing digital repository DRYAD once the manuscript is accepted for publication.

Reviewer #4

Q13: *I am not a fan of the 'leaky' cap expression present in title (but not in the abstract). Would not 'dynamic' cap be better? As opposed to 'stable' cap for Tmod. In any case, if this expression is important enough to make it into the title it should also appear in the abstract when the molecular model is discussed.*

A:

Calling Lmod “a dynamic cap” in our opinion is misleading, because Tmod capping is also dynamic in the sense that Tmod can be displaced from the pointed end (e.g. by Lmod). We prefer to keep the word “leaky” in the title. Therefore, we changed the abstract to include the same expression, as was recommended by the reviewer.

Q14: *Figure 4 should include the GFP-Lmod2 channel separately in grey to better illustrate the localisation of the wild type and mutant proteins. Does the sarcomere length change upon GFP-Lmod2 transfection?*

A:

We have now included panels into Figure 4A and 5A, showing the localization of GFP α-actinin separately. Sarcomere lengths were not significantly different between the groups, as indicated in the pertaining section of Materials & Methods.

Q15: *Of course, it would be nice to engineer a cardiomyocyte cell line with CRISPR that contains the L25G in Lmod to demonstrate the functional relevance, at least in the in vitro model. However, I can see this experiment being challenging in the current situation with limited access to the labs. Hence, I would not consider it essential.*

A:

We thank the reviewer for the suggestion. Creating a cell line with CRISPR will be a valuable approach for future studies, as the reviewer noted, once access to the labs and Cores are reopened.
