# Supplementary material for: Leiomodin creates a leaky cap at the pointed end of actin-thin filaments
Source: PLoS Biol. 2020 Sep 8;18(9):e3000848. doi: 10.1371/journal.pbio.3000848 (PMC7500696; doi:10.1371/journal.pbio.3000848)
Supplement: Attachment — Submitted filename: Response_to_reviewers_2_Tolkatchev.pdf [file pbio.3000848.s034.pdf]

Response to reviewers.

We are grateful to the reviewers for thorough examination of our manuscript and valuable suggestions and comments, which helped improve this paper. We have also changed the title, included files with raw data in supporting information, and made small editorial changes (corrected grammar errors and typos), as was requested in the editor's email.

Reviewer #1: *My comments were all addressed in the revised version.*

Reviewer #2:
Q1: *The only minor change that I would request is that the authors should add to the supplementary material some of the already recorded spectra that are mentioned in the response to the referee questions. E.g. it is mentioned that some line width reduction was observed in 15N HSQC spectra as a function of concentration of unlabelled peptide. It is also mentioned that some 13C correlated 3D spectra and some X-filtered NOESY spectra had only broadened peaks. It would be very useful if the 2D spectra and selected slices from the 3D spectra would be made available so that the interested reader could get a better idea of the dynamics of the system.*

A:

We have now added in the manuscript supplementary figures S12 Fig (entitled "Non-uniform broadening of cross peaks in 2D ^{15}N-HSQC spectra of ^{15}N/^{13}C-labeled αTM1a$_{1\text{-}14}$Zip upon addition of unlabeled Lmod2s1"), S13 Fig (entitled "Chemical exchange in the Lmod2s1/αTM1a$_{1\text{-}14}$Zip complex causes non-uniform line broadening in 3D ^{13}C-correlated spectra, thus imposing limitations on chemical shift assignment"), and S14 Fig (entitled "Examples of peak broadening in ^{13}C-correlated spectra of ^{15}N/^{13}C-labeled Lmod2s1 in the presence of excess αTM1a$_{1\text{-}14}$Zip."). These supporting figures provide information requested by the reviewer. References to the figures have been added in the manuscript on lines 677, 681 (S12 Fig), and 706 (S13 Fig, S14 Fig). The line numbers are given for the manuscript version with tracked changes.

Reviewer #3:

Q2: *The authors have addressed all my comments. About the release of the pdb structure of the complete model, I still think it needs to be made available as supplementary material, you can just state clearly that it is a model to avoid any confusion.*

A:

The pdb structure is now included in Supporting information (entitled "S3 Data") and a reference to this dataset has been added in the text of the manuscript (line 1263 of the manuscript version with tracked changes).

Reviewer #4

Q3. *The authors had uploaded Figure 4 twice and Figure 5 was missing from the revised version, but there was no change requested on Figure 5.*

A:

The correct Figure 5 has been uploaded.
